# The effect of perceived social support on psychological distress and life satisfaction among Nepalese migrants in Japan

**DOI:** 10.1371/journal.pone.0246271

**Published:** 2021-02-26

**Authors:** Januka Khatiwada, Basilua Andre Muzembo, Koji Wada, Shunya Ikeda

**Affiliations:** 1 Department of Public Health, School of Medicine, International University of Health and Welfare, Narita, Japan; 2 Graduate School of Public Health, International University of Health and Welfare, Otawara, Japan; Nepalese Society of Community Medicine, NEPAL

## Abstract

**Background:**

The world is becoming individualized due to modernization. International migration is one of the factors that lead to family dissociation and a lack of social support. Social support is viewed as a crucial factor that contributes to psychological well-being and satisfaction with life among migrants. However, very little is known about the impacts of social support on psychological distress and satisfaction with life among migrants. Therefore, we conducted this study to assess the association of perceived social support with psychological distress and satisfaction with life among Nepalese migrants, and we evaluated the factors associated with receiving social support.

**Methods:**

This was a cross-sectional study conducted with a convenience sample of Nepalese migrants (N = 249) living in Tokyo. Self-administered online questionnaires were distributed using social networks and chain referral methods. The measures included the Multidimensional Scale of Perceived Social Support, the General Health Questionnaire, and Satisfaction with Life Scale. Descriptive analysis, Pearson’s correlation analysis, and multiple linear regression analyses were performed using SPSS ver. 25.

**Results:**

The mean (SD) age of the respondents was 31.8 years old (7.3). The family, friends, and significant others subscales of the multidimensional scale of perceived social support were negatively correlated with psychological distress (p<0.01). The family subscale was positively correlated with satisfaction with life (p<0.05), and the friend and significant others subscales were positively correlated with satisfaction with life (p<0.01). Social support from the family was significantly and negatively associated with the type of visa (Beta = -0.160, p = 0.049), and marital status was negatively associated with support from significant others (Beta = -0.175, p = 0.024).

**Conclusion:**

Social support from family, friends, and significant others was found to be influential in decreasing psychological distress and increasing satisfaction with life among Nepalese migrants in Tokyo. Strengthing social support system through the expansion of interpersonal network may help minimize the psychological distress

## Background

The world is becoming individualized due to modernization [1]. Social connections, emotional support, and family relationships are being replaced by self-centered, personalized, and egocentric modes of life. International migration is a worldwide phenomenon and is one of the factors that contribute to family dissociation, low levels of emotional support, and low levels of social support [2]. People migrate to foreign countries temporarily and permanently for various reasons, such as seeking employment, studying abroad, and searching for a better life. Migration can be beneficial for both migrants and host countries in terms of economic prosperity, cultural diversity and exchange, and enhancement of knowledge and skills [3]. On the contrary, migrants have an increased likelihood of problems related to their health, psychological state, family dissociation, and isolation [2]. Social support is viewed as a crucial factor that contributes to well-being even among individuals with a high level of stress [4]. Social support is a multifaceted concept that has various indicators, such as the frequency of contact with families, friends, spouses, or partners, the level of affinity in giving and receiving support; supportive functions; and perceived satisfaction with life [5]. Social support is a fundamental need for people who migrate to foreign countries, as it is related to their psychological well-being. [6]. Receiving social support helps to enhance psychological well-being [7]. According to World Health Organization (WHO), a lack of social support is associated with not only an increase in mortality and morbidity but also an increase in psychological distress and a decrease in well-being [8]. However, few studies have examined how social support affects migrants’ level of psychological distress.

Social support has also been associated with satisfaction with life among migrants. Korean elderly immigrants reported higher life satisfaction if they get higher levels of social support [9]. A lack of social support may contribute to creating feelings of loneliness and distress that may lead to difficulties in maintaining relationships and avoiding social situations due to the fear of exclusion [10].

Japan has become one of the destinations for Nepalese migrants. About 87,148 Nepalese were residing in Japan in 2018 [11]. They were specifically in cook, dependent, skilled labor, high-skilled personals, students, and other visa statuses. A previous study reported that Nepalese migrants’ health is deteriorating in Japan due to language barriers, lack of awareness, and no or discontinuation of payment of national insurance premium [12]. Another study documented that lack of access to social support was a determinant of mental health well-being among the migrants in Japan [13]. Limiting themselves on securing their financial stability by working many hours and managing work-study balance lacks time for connecting with family and friends in Nepal and the Nepalese community in Japan. It may affect their psychological well-being and satisfaction with life. However, relatively few studies have measured the impacts of social support on psychological distress and satisfaction with life among migrants. Therefore, we conducted this study to assess the association of perceived social support with psychological distress and satisfaction with life and the factors associated with receiving social support among Nepalese migrants living in Tokyo.

## Methods

### Study design and area

This was a cross-sectional study measuring the impact of perceived social support on psychological distress and satisfaction with life among Nepalese migrants. The study was conducted in Tokyo because majotities of Nepalese (26,157 as of 2018) migrants live in Tokyo compared to other prefectures. They were cooks in Indo-Nepali restaurants, dependents, students, business owners, permanent residents, and others. Dependents and students mostly work in hotels, restaurants, and food packaging companies. The survey was conducted between July and August 2019.

### Sampling method and participants

A convenience sampling method was used to recruit participants. The sample size was calculated using the z-test. Though the study estimated the required sample using a 95% confidence interval and a 5% margin of error within a reference population of Nepalese 26,157 in Tokyo, individual participants’ selection was made conveniently. A self-administered computer-assisted online questionnaire was used for those who could complete the measures by themselves. We disseminated the questionnaire to 380 participants who met the eligibility criteria (mentioned in different sections below) using Social Networking Service (SNS) and chain referral methods. We obtained 272 questionnaires. We had to exclude 23 questionnaires due to missing data; thus, data from 249 participants were analyzed.

We applied the inclusion criteria as being aged 18 to 60 years, Nepali citizens, and live continuously in Japan for at least six months. We excluded the participants who couldn’t read and write in Nepali.

### Study tools and variables of the study

The Multidimensional Scale of Perceived Social Support (MSPSS) was used to measure social support. The MSPSS measures perceived social support from three sources: family, friends, and significant others [14]. This Likert scale consists of 12 items ranging from 1 (very strongly disagree) to 7 (very strongly agree). Total scores range from 12 to 84, and higher scores represent greater perceived social support. The Nepali version of the MSPSS was used herein. This scale was previously translated to Nepali, and its validity has been assessed in the Nepalese population and Nepalese migrants [12, 15].

Psychological distress was measured using the General Health Questionnaire (GHQ-12), which contains 12 questions. The responses of each item were measured on a 4-point Likert scale ranging from 0 (not at all) to 3 (much more) for negative items and 0 (better than usual) to 3 (very worse than usual) for positive items. We reversely recoded negative items to resemble them with positive items. We used the Nepali version of the GHQ, which has been validated among Nepali respondents [16]. A lower score indicates lower levels of psychological distress.

The Satisfaction with Life Scale developed by Diener, Emmons, Larsen & Griffin [17] was used to measure the satisfaction level of the respondents. The scale consists of five items with a seven-point scale ranging from 1 (strongly disagree) to 7 (strongly agree). Sample items included ’In most ways, my life is close to my ideal’ and ’If I could live my life over, I would change almost nothing.’ The satisfaction with life scale used herein was previously validated among Nepali respondents [18]. A higher score indicates higher levels of satisfaction.

### Data analysis

The data were analyzed using SPSS software ver. 25 (IBM, NYC). Descriptive analysis was performed using mean (SD) for age and frequencies and percentages for the following variables: sex, age, marital status, ethnicity, educational status, visa/legal status, length of stay in Japan, and language proficiency. We divided sex as male and female; age as 30 and below, 31 to 40, and 41 and above; marital status as married, unmarried, and others; ethnicity as Brahmin/ Kshetri/Thakuri, Janajati, Dalit, and others referring to the caste division of Nepal Government applied in census 2011 [19]; educational status was divided as primary, secondary, and university level; visa/legal status as a dependent, student, skilled labor, engineer/specialist in humanities/international services, permanent resident/spouse or child of permanent resident, and others; length of stay as 0–5 years, 6–10 years, 11–15 years, and 16 years above; and language proficiency as not at all, basic, intermediate, and advanced level. Pearson’s correlation analysis was employed to analyze the associations between perceived social support and psychological distress and satisfaction with life. Multiple linear regression analysis was performed to determine which sociodemographic factors were associated with perceived social support. In the multiple linear regression, we treated Likert scale variables as continuous variables after summing up each subscale’s total score. The total score for family, friends, and significant other subscales of MSPSS ranged from 4 to 28. We also checked the multicollinearity among the predictors’ variables before running the analysis.

### Reliability

Cronbach’s alpha coefficient was computed to assess the internal reliability of the study tools. Cronbach’s alpha coefficient for the total MSPSS was 0.96. The family, friends, and significant other subscales had Cronbach’s alpha values of 0.94, 0.92, and 0.94, respectively. We also measured the reliability of psychological distress (GHQ) and satisfaction with life (SL) measures. The Cronbach’s alpha coefficient for the GHQ and the SL scale was 0.88 and 0.80, respectively. The results indicate that the overall reliabilities of the MSPSS, GHQ, and SL were quite good and comparable with each other.

### Ethical approval

The Ethical Committee of the International University of Health and Welfare, Graduate School, Japan approved the research protocol (Approval Number 18-Im-014). Data were collected after obtaining written informed consent from each participant. The questionnaire was developed online with an attachment of an informed consent form with an explanation of objectives, loss/benefit for the respondents, and the expected outcome of the research. The participants who agreed to participate were asked to provide their consent with their name before filling in the questionnaire.

## Results

### General characteristics

Table 1 shows the general characteristics of Nepalese migrants living in Tokyo who participated in this study (N = 249). The mean (SD) age of the respondents was 31.8 (7.3) years old. Among all the respondents, 49.0% were aged 30 and below. Approximately 67% of them were males. About 75.9% of the respondents were married, and 76.3% belonged to the Brahmin/Kshetri/Thakuri castes. About 75.5% had a university-level education. Approximately 57.8% of respondents reported that they had an intermediate level of Japanese language proficiency. About 27.3% had an engineer/specialist in humanities/international services visas, and 52.2% had been living in Japan for fewer than five years.

**Table 1:** General characteristics of Nepalese migrants in Tokyo.

Variables	N = 249	%
**Age Mean (SD)**	31.8 (7.3)	
30 and below	122	49.0
31–40 years	98	39.4
41 and above	29	11.6
**Gender**		
Male	166	66.7
Female	83	33.3
**Marital status**		
Married	189	75.9
Unmarried	57	22.9
Others	3	1.2
**Caste**		
Brahmin/Kshetri/Thakuri	190	76.3
Janajati	51	20.5
Dalit	4	1.6
Others	4	1.6
**Education**		
Primary level	6	2.4
Secondary level	55	22.1
University level	188	75.5
**Japanese Language Proficiency**		
Not at all	1	0.4
Basic level	76	30.5
Intermediate level	144	57.8
Advanced level	28	11.2
**Types of visa**		
Dependent	54	21.7
Students	58	23.3
Skilled labor (Cook)	32	12.9
Engineer/Specialist in Humanities/International Services	68	27.3
Permanent resident/Spouse or child of permanent resident	19	7.6
Others	18	7.2
**Length of stay in Japan**		
0–5 years	130	52.2
6–10 years	70	28.1
11–15 years	33	13.3
16 and above	16	6.4

The family, friends, and significant other dimensions of the MSPSS subscales were negatively correlated with psychological distress (p<0.01). The family subscale of the MSPSS was positively correlated with satisfaction with life (p<0.05), and the friend and significant other subscales of the MSPSS were positively correlated with satisfaction with life (p<0.01) (Table 2).

**Table 2:** Correlation matrix for the family, friends, and significant others subscales, psychological distress, and satisfaction with life.

Variables	MSPSS Family	MSPSS Friends	MSPSS Significant Others	Psychological distress	Satisfaction with life
MSPSS Family	1				
MSPSS Friends	.637**	1			
MSPSS Significant Others	.750**	.714**	1		
Psychological distress	-.211**	-.308**	-.322**	1	
Satisfaction with life	.135*	.260**	.248**	-.480**	1

* P value < 0.05

** P value < 0.01

Table 3 presents the result of the multiple linear regressions. Perceived social support from the family was significantly negatively associated with the type of visa (Beta = -0.160, p = 0.049). No predictors were significantly associated with the friends’ subscale of the MSPSS. Marital status was negatively associated with the significant others subscale of the MSPSS (Beta = -0.175, p = 0.024).

**Table 3:** Multiple linear regression between the family, friends and significant others subscales of the MSPSS and sociodemographic characteristics.

Variables	Unstandardized Coefficients		Standardized Coefficient	*P value*
	B	SE	Beta	
**MSPSS family**				
Gender	-0.095	0.112	-0.064	0.399
Age	0.006	0.059	0.010	0.917
Marital status	-0.162	0.118	-0.106	0.173
Caste	0.089	0.080	0.073	0.265
Education	0.129	0.092	0.090	0.164
Length of stay in japan	-0.029	0.063	-0.038	0.639
Visa type	-0.074	0.037	-0.160	**0.049**
**MSPSS friends**				
Gender	0.133	0.127	0.080	0.295
Age	0.017	0.066	0.023	0.803
Marital status	-0.139	0.134	-0.081	0.299
Caste	0.069	0.090	0.051	0.440
Education	0.098	0.104	0.061	0.347
Length of stay in japan	0.010	0.071	0.012	0.883
Visa type	0.010	0.042	0.019	0.816
**MSPSS significant others**				
Gender	0.082	0.127	0.048	0.518
Age	-0.007	0.067	-0.010	0.918
Marital status	-0.304	0.134	-0.175	**0.024**
Caste	0.048	0.090	0.034	0.599
Education	0.143	0.104	0.088	0.171
Length of stay in japan	0.038	0.071	0.043	0.596
Visa type	-0.024	0.042	-0.047	0.564

## Discussion

The major finding of the study was 1) the perceived social support was influential to psychological distress and satisfaction with life of migrants, and 2) type of visa might be a determinant for receiving support from the migrant’s family and marital status for receiving support from significant others.

Perceived social support was found to be significantly correlated with psychological distress and satisfaction with life; in particular, the family, friends, and significant other subscales of the MSPSS were reported to be negatively correlated with psychological distress and positively correlated with satisfaction with life. Another study conducted with individuals with drug addiction revealed a positive correlation between social support and satisfaction with life [20]. Social support helps reduce problematic behaviors, thus enhancing their adjustment and reducing adverse effects on their psychological state [21].

This study revealed that higher levels of social support lead to lower levels of psychological distress. This finding indicates that social support could be used to maintain a stable psychological state. Support from family, friends, and significant others helps to prevent psychological distress. Relatively few studies have been conducted to analyze the effect of perceived social support on the psychological distress of migrants. Support from family, friends, and significant others may provide emotional strength to better adapt and adjust to new places and new cultures.

Social support from family, friends, and significant others is also significantly correlated with satisfaction with life among migrants. We couldn’t find the study about the association of social support with satisfaction with life among migrants; however, this result is consistent with another study conducted with university students [22–24]. Sharing feelings with others makes people feel better and makes them treasure each moment with happiness. It also prepares people to cope with every problematic situation and stress [25].

We also conducted multiple linear regression to identify which sociodemographic factors are associated with receiving support from family, friends, and significant others. The results revealed that perceived social support from the family was significantly negatively associated with the type of visa. Migrants with dependent visas, student visas, and cooking visas might lack family support compared to migrants with other types of visas. We could not find other studies with findings that are consistent with ours. The reason for this finding might be that the students have less time to be connected with families in their home countries. Migrants with dependent and cooking visas tend to get less support from family, which may be due to their long and unmatched working hours, leaving them less time to devote to their family.

Similarly, marital status was negatively associated with the significant other subscale of the MSPSS. Married migrants tend to receive lower levels of support from significant others. This can be attributed to the practice of restrictions and limitations associated with married life that leads migrants to obtaining support from others rather than spouses. A significant other is not defined in the questionnaire, and thus, respondents could have thought about anyone as a significant other in their lives. Therefore, these results may not be generalizable.

This study has some limitations. We only collected data in Tokyo. Collecting data in other prefectures where Nepalese migrants live would be a more holistic approach to obtain more generalizable results. As the study results indicate, Nepalese migrants living in Tokyo may not represent all Nepalese migrants. The result might differ in other prefectures, as working conditions, visa types, and the local government’s facilities are different in different prefectures. Another limitation was convenience sampling may not provide a representative estimation of the sample population as it may lead to inherent bias.

## Conclusion

Social support from family, friends, and significant others was found to significantly decrease psychological distress and increase satisfaction with life among Nepalese migrants in Tokyo. Receiving support from family, friends, and significant others might be essential for the psychological well-being of migrants. Type of visas differs the support from family and marital status to get support from significant others. Strengthening the social support network through the expansion of social involvement may help minimize the rate of psychological distress among the migrants.
